# Compact and Thermosensitive Nature-inspired Micropump

**DOI:** 10.1038/srep36085

**Published:** 2016-10-31

**Authors:** Hyejeong Kim, Kiwoong Kim, Sang Joon Lee

**Affiliations:** 1 Center for Biofluid and Biomimic Research, Department of Mechanical Engineering, Pohang University of Science and Technology (POSTECH), Pohang, 790-784, South Korea

## Abstract

Liquid transportation without employing a bulky power source, often observed in nature, has been an essential prerequisite for smart applications of microfluidic devices. In this report, a leaf-inspired micropump (LIM) which is composed of thermo-responsive stomata-inspired membrane (SIM) and mesophyll-inspired agarose cryogel (MAC) is proposed. The LIM provides a durable flow rate of 30 μl/h · *cm*^2^ for more than 30 h at room temperature without external mechanical power source. By adapting a thermo-responsive polymer, the LIM can smartly adjust the delivery rate of a therapeutic liquid in response to temperature changes. In addition, as the LIM is compact, portable, and easily integrated into any liquid, it might be utilized as an essential component in advanced hand-held drug delivery devices.

During last decades, academic and biomedical interests have focused on the development of practical microfluidics, such as portable drug delivery systems, simple diagnosis or point of care (POC) devices, to improve the quality of life^1^,^2^,^3^,^4^,^5^,^6^,^7^. An automatic insulin-dosing pump is one of typical examples inevitable for diabetic patients to prevent sudden hyper/hypoglycemia due to irregular taking of medicine^2^,^3^,^8^. Although various types of insulin-dosing devices have been introduced, there are still remaining parts to be improved^9^. Conventional drug-delivery pumps are bothersome to handle due to their bulky size and the needs of external power supply. Thus, they are required to be miniaturized and integrated with several fluidic components without using an external macro-scale power source or controller. To meet these demands, various types of non-mechanical micropumps utilizing capillary force, surface tension, evaporation, vacuum-driven, and paper-based devices, have been developed^4^,^10^,^11^,^12^,^13^. However, most of them still have technological limitations of single-use, short-term operation, or needs of additional integration with a dedicated flow controller. Among them, the evaporation-driven micropump can meet the demand of continuous flow supply for a long time. Some evaporative micropumps with various flow control methods and their specifications are summarized in Table 1^14^,^15^,^16^,^17^,^18^,^19^,^20^,^21^,^22^,^23^,^24^. Duration of flow supply is from few hours^17^,^19^,^21^,^22^ to few days^15^,^20^. The flow rates are ranged from 0.002 μl/min for passive pumping to 3.7 ml/min for actively controlled evaporation pumping. For active control of the evaporation pumps, electrodes^14^ or surface acoustic waves^23^ are especially utilized, but most of them are passively controlled by temperature changes. In addition, since most of them are usually dedicated to a specific microfluidic channel, they cannot be applied to other flow devices^14^,^16^,^17^,^18^,^22^,^23^,^24^.

To resolve those technological limitations, we derive a novel solution bioinspired by plant leaf transpiration. Actually, a plant leaf is a kind of powerful hydraulic pump, which continuously ascends water from the roots to the leaves against gravity without any mechanical moving parts^25^. Inside a plant leaf, nanostructures found in the mesophyll cell wall create a high negative water potential of approximately −2 MPa^26^. In plant hydraulics, the water flow rate (*q*) of a plant can be expressed as follows: 
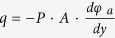
, where *φ*_*a*_ is the water potential at a certain point of *a*, and *P* and *A* represent the water permeability and surface area of water path inside the plant, respectively. The potential difference Δ*φ* which is a driving force of water transport can be expressed by the difference between *φ*_*soil*_ and *φ*_*leaf*_. Water absorbed from the roots is transported via various pathways to air spaces inside the leaves (Fig. 1a). Water molecules in the leaves then evaporate through tiny stomatal pores. A stoma opens and closes its aperture to function as a multisensory flow regulator in response to various environmental conditions^25^.

In this study, a novel leaf-inspired micropump (LIM) was developed by mimicking the transpiration functions of a plant leaf. The LIM (Fig. 1b) is a non-mechanical hydraulic pump aided by a thermo-sensitive flow control. It is composed of a thermo-sensitive stoma-inspired membrane (SIM) and a macroporous mesophyll-inspired agarose cryogel (MAC). As water molecules in the SIM evaporate into the air, the highly water permeable MAC compensates the water loss against gravity. The LIM guarantees to supply a continuous and slow flow rate of 30 μl/h·*cm*^2^ for more than 30 h at room temperature. This is a multiple-use pumping system applicable to any hydrophilic liquid including therapeutic agents. Using this system, we can resolve the technical limitations of single-use or short-term use encountered in capillary-based or paper-based micropumps^4^,^10^,^11^,^12^,^13^. The LIM can also cope with the thermally sensitive flow rate of conventional evaporative micropumps, whose flow rate is passively determined on the basis of temperature, by using thermo-sensitive hydrogels, poly(*N*-isopropylacrylamide) (PNIPAAm) (Table 1)^14^,^15^,^16^,^17^,^18^,^19^,^20^,^21^,^22^,^23^,^24^. The LIM can control the timing of water pumping by setting a specific local critical solution temperature (LCST, 33 °C), which is around the surface temperature of human body. In addition, as the LIM is compact, portable, and easily applied to any liquid. This study may be used as a basis for novel designs of advanced healthcare devices, such as POC or drug-delivery system.

## Results

### Leaf-inspired evaporative micropump

**Figure 2:**
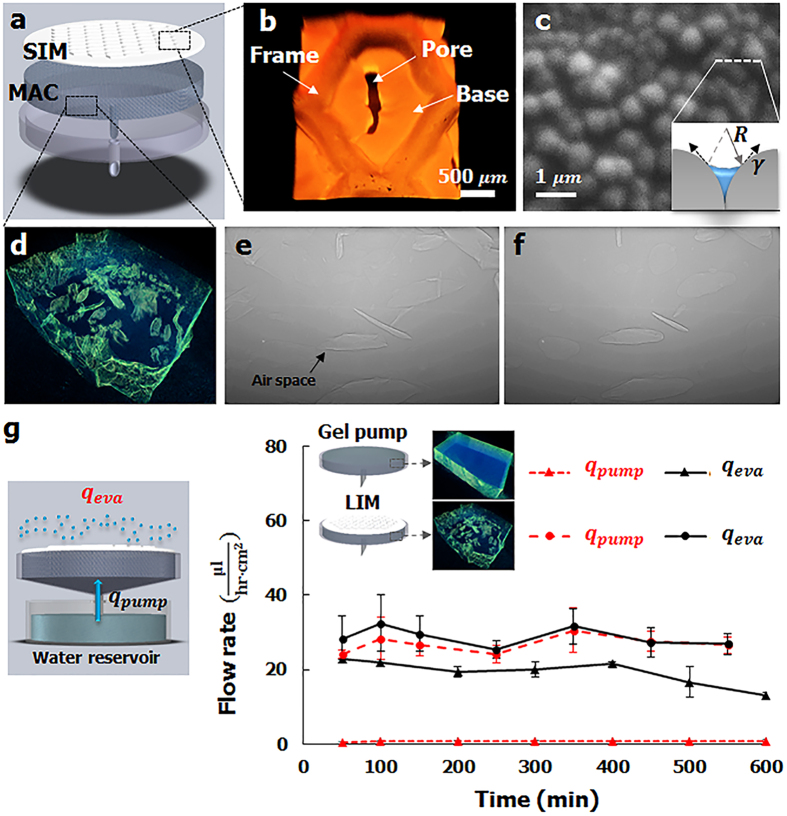
Components of the LIM and its pumping performance at room temperature. (**a**) The LIM is composed of two parts, namely, SIM and MAC. (**b**) Confocal image of the SIM, which consists of slit-shaped pores, a base part, and a thick frame. (**c**) A typical SEM image of the SIM surface. Rounded grooves of a hundred-nanometer scale were formed. Water molecules accumulate in the grooves and the air-water interface generates negative Laplace pressure. (**d**) 3D structure of the macroporous MAC visualized through an X-ray micro-imaging technique. (**e**) Initial state of the dehydrated MAC. (**f**) As water was supplied to the MAC, air spaces inside the gel were rapidly filled with water. (**g**) Comparison of the evaporation flow rates and pumping flow rates of the agarose gel pump and the LIM.

The LIM is composed of a thermo-sensitive hydrogel SIM and a macroporous hydrogel MAC (Fig. 2a). Similar to plant leaves, the proposed pump works on the basis of the potential difference Δφ between *φ*_*source*_ and *φ*_*SIM*_. As water molecules in the SIM evaporate into the air, *φ*_*SIM*_ decreases to a negative value, and Δ*φ* between *φ*_*source*_ and *φ*_SIM_ provides the driving force for pumping.

The SIM comprises three parts, namely, slit-shaped pores, a relatively thin base, and a thick frame (Fig. 2b)^27^. On the surface of the SIM, water molecules accumulate in the rounded grooves of a hundred-nanometer scale and form air-water interfaces (Fig. 2c). Water under a nanometer-scale curved surface develops a highly negative Laplace pressure expressed as follows: 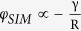, where γ is the surface tension of deionized water (7.28 × 10^−8^*MPa*·*m*) and R is the radius of the curvature of the air-water interface on the grooves. This negative suction pressure is the main driving power of the LIM to facilitate continuous water pumping.

The polydimethylsiloxane (PDMS) body is filled with MAC and the tip of the attached needle is exposed to a liquid source. The MAC is composed of a highly hydrophilic agarose cryogel with a porosity of 5.57%. It consists of a macroporous hierarchical structure containing nanoscale pores with an average size of 1.58 nm and microscale pores with an average size of 106.5 μm (Fig. 2d)^28^. The MAC functions as a water transporter from the water source to the evaporative surface because the macroporous structure exhibits high water permeability. As water is supplied to the dehydrated MAC, the air spaces inside the gel are rapidly filled with water within a few seconds (Fig. 2e,f; Movie S1). Therefore, the MAC stably compensates for the water loss in the SIM if the tip of the needle touches the water source.

To analyze the pumping performance, the LIM was installed at the water reservoir (Fig. 2g). To minimize the flow resistance in the passage from the water reservoir to the pump during the pumping process, the needle of the LIM directly touched the water surface of the reservoir. *φ*_SIM_ decreased to a negative value as water evaporates; as such, water was pumped up from the reservoir where water potential was zero. The evaporation rate and the pumping rate of the LIM remained at 28.52 ± 3.47 and 29.47 ± 4.47μl/min·cm^2^ for 20 h, respectively, (Fig. 2g, circle dots). The almost similar evaporation rate and pumping rate indicated that the water loss caused by evaporation was stably compensated by water pumped by MAC. On the other hand, water permeability was reduced when the pump body was filled with normal agarose gel containing nanoscale pores. Thus, the evaporation rate gradually decreased from 20.72 ± 1μl/min·cm^2^ to 13.14 ± 1μl/min·cm^2^ (Fig. 2g, triangle solid line) because water loss cannot be sufficiently compensated by water pumping (Fig. 2g, triangle dotted line). Therefore, the highly water-permeable agarose cryogel should be used to pump enough water from the reservoir (Figure S2). The evaporation rate of the LIM was 37% higher than that of the agarose gel pump. This faster evaporation rate results from the bumpy surface of the SIM by which the area of evaporative surface is increased (Fig. 2b,c).

### Thermal response of the LIM

**Figure 3:**
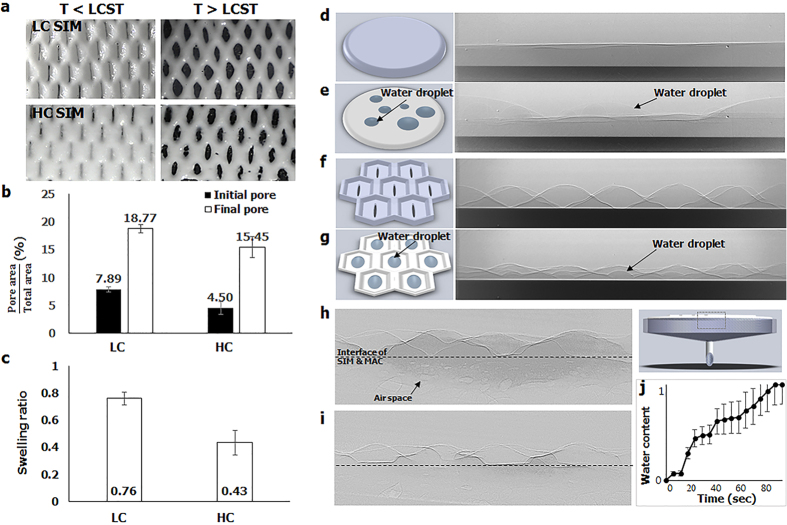
Thermal responses of the LIM. (**a**) Two different SIMs were fabricated with a low crosslinker concentration (LC) and a high crosslinker concentration (HC) to analyze the thermal response of the SIMs. The LC SIM was composed of a clear frame, a base, and pore parts. The HC SIM consisted of a base and pore parts with a blurred frame. As the membranes were heated at a temperature higher than LCST, the material shrank and the slit pores opened in an elliptic shape. (**b**) Variations in the pore surface area of the SIMs. (**c**) Comparison of the mass swelling ratios of the two SIMs. (**d**) Initial state of the flat PNIPAAm membrane. (**e**) Final state of the flat PNIPAAm membrane after it was heated at a temperature higher than LCST. (**f**) Initial state of the SIM. (**g**) Final state of the SIM after it was heated at a temperature higher than LCST. (**h**) Initial state of the SIM-applied MAC. (**i**) Final state of the SIM and the MAC after they were heated at a temperature higher than LCST. (**j**) Temporal variation of the normalized relative water content inside the MAC.

As the stomata open and close their apertures in response to environmental stimuli, the SIM exhibits a gating function because of cyclic temperature changes (Fig. 3a). The pumping performance largely depends on evaporation rate which is highly relevant to environmental conditions where the evaporative surface is exposed. For instance, the evaporation rate exponentially increases with the increase of temperature, and linearly decreases with the increase of relative humidity (Supporting information 1). In this study, we mainly focus on the effect of temperature variation on the pumping performance of the proposed pump. Volumetric shrinkage occurs with the extrusion of water molecules when the thermo-responsive PNIPAAm polymer is heated to a temperature higher than LCST (33 °C). The water extrusion and absorption through the polymer network arises from the wettability change of PNIPAAm. The PNIPAAm becomes hydrophobic at temperatures higher than the LCST, and the material become hydrophilic at the temperature lower than the LCST^27^.

To analyze the structural variations in the SIMs in response to thermal stimuli, two SIMs with different crosslinker concentrations were fabricated: 0.143 wt% of low crosslinker concentration (LC) and 0.429 wt% of high crosslinker concentration (HC). The LC SIM was composed of a clear frame, base, and pore parts. However, the frame of the HC SIM was blurred (Fig. 3a). The pores opened as the polymer network shrank when the edges of the SIMs were anchored and heated to a temperature higher than LCST (Movie S2). The area of the pores of the LC SIM increased from 7.89% to 18.77% of the total surface area, and that of the HC SIM increased from 4.5% to 15.45% of the total area (Fig. 3b). The LC SIM yielded a higher swelling ratio and contained a higher amount of water inside the polymer network because the low concentration of the crosslinker induced a low degree of polymerization (Fig. 3c, Fig. S3).

Water molecules, extruded from a flat PNIPAAm membrane by heating, randomly accumulated on the membrane surface (Fig. 3d,e; Movie S3). However, due to presence of grooves on the SIM, the extruded water droplets exactly accumulated just at the pores of the membrane (Fig. 3f,g; Movie S4). When the SIM was applied to the MAC, the MAC absorbed the accumulated water droplets from the membrane. A typical X-ray image (Fig. 3h) shows the side view of the interface between the SIM and the MAC (dotted square of inset). Air spaces of various shapes and sizes (light gray) were distributed inside the MAC. After the components were heated, the air spaces were replaced with water extruded from the SIM (Fig. 3i; Movie S5). The temporal variation of the normalized relative water content inside the MAC was examined on the basis of the measured intensity data by applying Beer-Lambert’s law (Fig. 3j)^28^,^29^. As water extruded from the SIM was absorbed by the MAC, the water content in the MAC abruptly increased during heating.

### Thermo-controlled pumping performance

**Figure 4:**
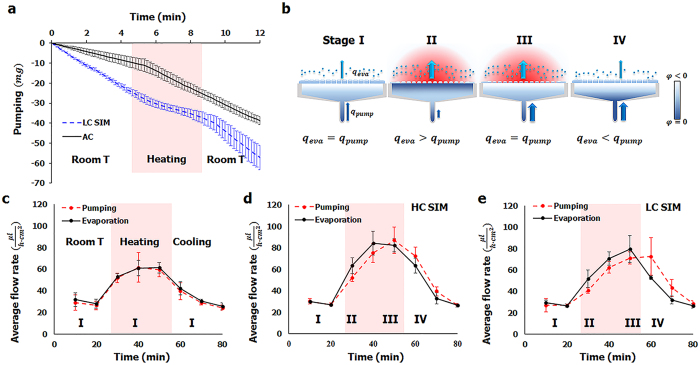
Thermo-controlled pumping performance of the micropump. (**a**) Comparison of the mass loss in the water reservoir pumped by the AC pump and the LIM with the LC SIM. The pump system was heated from room temperature to a temperature higher than the LCST of PNIPAAm for 3 min (shaded region) and then cooled to room temperature, again. (**b**) Four stages of the pumping process: I, initial condition of pumping at room temperature; II, early stage after heating at a temperature higher than LCST; III, after heating at for a given period; and IV, cooling again to room temperature. The average evaporation rates and the pumping rates of the AC pump (**c**), the LIM with HC SIM (**d**), and the LIM with LC SIM (**e**) were compared to demonstrate these four stages.

A thermo-controlled pumping performance of the LIM was demonstrated by determining the amount of water loss in the reservoir. The AC pump, composed of agarose cryogel without the SIM, was used as a control group. The pumps were heated from 28.6 ± 0.2 °C to 45.45 ± 0.7 °C at an increasing rate of 3.87 ± 0.34 °C/min and then cooled to room temperature at a decreasing rate of −1.47 ± 0.27 °C/min. Before the AC pump was heated, the water loss rate was 2.10 ± 0.8mg/min (Fig. 4a). After the pump was heated, the water loss rate then increased to 4.17 ± 1.45 mg/min. By contrast, the water loss rate decreased from 5.3 ± 2.37 mg/min to 2.48 ± 1.1 mg/min by heating when the LIM was combined with LC SIM. The water loss rate of the LIM increased again to 6.32 ± 2.98 mg/min by cooling to room temperature.

The mechanism of thermo-controlled water pumping can be further explained by the following consideration (Fig. 4b). The evaporation rate in the SIM and the pumping rate from the water reservoir can be divided into four stages on the basis of temperature variation and duration. In the first stage, the evaporation rate and the pumping rate are almost the same when temperature is lower than LCST because evaporation-induced water loss in the SIM was compensated by the water pumped from the reservoir (*q*_*eva*_ = *q*_*pump*_). In the second stage, when temperature is higher than LCST, the evaporation rate increases (see Supplementary Section 1 for details). At the same time, the *φ*_*MAC*_ increases to reduce Δ*φ* between the MAC and the water reservoir because the MAC absorbs water extruded from the SIM. Therefore, the water pumping rate decreases and becomes smaller than the evaporation rate (*q*_*eva*_ > *q*_*pump*_). In the third stage, when temperature maintain higher than LCST, all of the absorbed water in the MAC evaporates and *φ*_*MAC*_ decreases. Thus, the evaporation rate and the pumping rate become similar again (*q*_*eva*_ = *q*_*pump*_). Finally, when temperature decreases lower than LCST, the surface of the SIM becomes hydrophilic and absorbs water. Therefore, the water pumping rate is the sum of the evaporation rate and the water absorption rate by the SIM (*q*_*eva*_ < *q*_*pump*_). More detailed explanation is described in Supporting Information 1.3.

To demonstrate the above four stages, the evaporation rate and the pumping rate were measured, while the pumping system was heated up and cooled down in the same manner. For the AC pump without the SIM, the evaporation rate and the pumping rate increased/decreased simultaneously as temperature increased/decreased (Fig. 4c). Stage I was maintained during the whole thermo-controlled processes. By comparison, the four stages were distinguished clearly when the HC SIM and the LC SIM were applied to the MAC (Fig. 4d,e). In both cases, the evaporation rate and the pumping rates were almost the same before the heating process was initiated (stage I). As heating proceeded, the evaporation rate increased and exceeded the pumping rate (stage II). As heating was maintained for about 20 min, the intersection points of two lines appeared when the pumping rate and the evaporation rate became equal (stage III). In stage IV, the pumping rate exceeded the evaporation rate. As the final outcome, water pumping was delayed from the time of water evaporation. Nevertheless, the total areas under the curves of the evaporation rate and the pumping rate were the same, with a 1% error bound in all of the three cases. It indicates that the total amount of evaporated water was totally compensated by water pumping.

A higher amount of water evaporated in the LIMs than in the AC pump (Fig. 4c–e; Figure S4a). This finding was mainly attributed to the bumpy surface of the SIM which increased the total area of the evaporative surface (Fig. 2g). The timing of the pumping of the LC LIM was more delayed than that of the HC LIM (Fig. 4d,e; Figure S4b). At this time, shifting is a result of a prolonged stage II in the LC LIM compared with that in the HC LIM. A longer time was necessary to evaporate a sufficient amount of water and thus generate a high negative potential to uptake water from the reservoir because the LC SIM extruded more water than the HC SIM did (Figure S3).

### Integration into microfluidics

**Figure 5:**
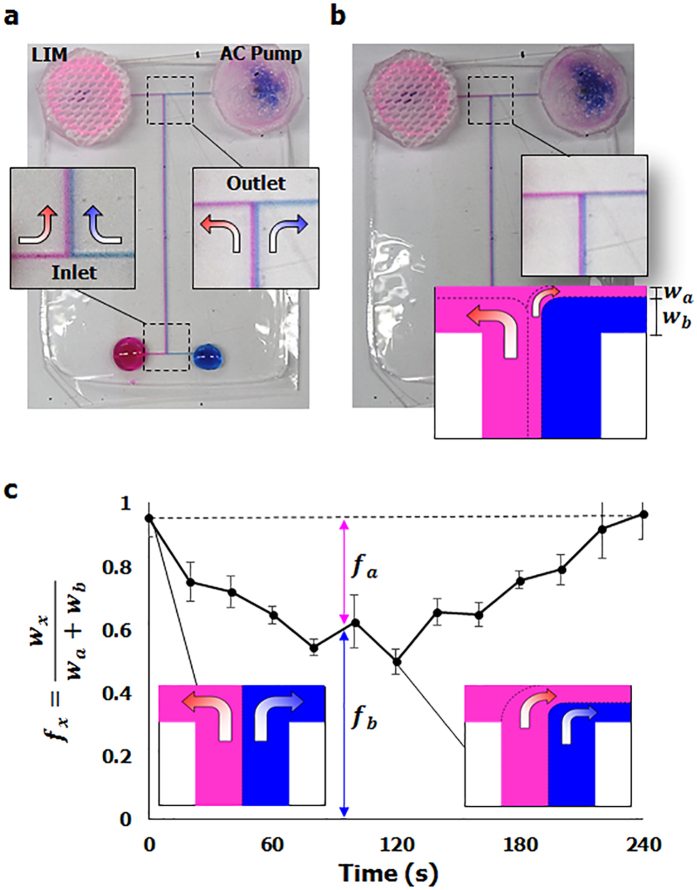
LIM and AC pump are integrated into an H-shaped channel. (**a**) At room temperature, Rhodamine-B and methylene blue solutions were separated into two opposite directions at the crossroads; the pink solution moved to the LIM, and the blue solution went to the AC pump. (**b**) After heating two pumps to a temperature higher than LCST, the slit-shaped pores of the SIM were opened. Afterward, the pink and blue solutions were drawn toward the AC pump. *w*_*a*_ and *w*_*b*_ are the widths of Rhodamine-B and methylene blue solution passing through the channel of the AC pump side. (**c**) The relative flow rates of the solutions at the channel of the AC pump side were evaluated after heating the two pumps for 120s and cooling down to room temperature afterward. The values of *f*_*a*_ and *f*_*b*_ indicate the relative flow rates of Rhodamine-B solution and methylene blue solution, respectively.

The compact and portable LIM can be easily applied to various microfluidic devices. To demonstrate the strong potential of the thermo-controlled pumping of the LIM, the LIM and the AC pump were adaptively integrated into an H-shaped channel (Fig. 5, Figure S5). Rhodamine-B solution, which appears pink, was dropped at the inlet of the LIM side; methylene blue solution was dropped at the opposite side inlet of the AC pump to distinguish the performances of the two pumps. As water evaporated from the pumps, the two dye solutions were drawn from the inlets of the channel to the outlet sides of the pumps. The relative flow rate of solution *x* moving through the channel of AC pump side was quantitatively evaluated using the definition of *f*_*x*_ = (*w*_*x*_)/(*w*_*a*_ + *w*_*b*_), where *w*_*a*_ and *w*_*b*_ are the widths of Rhodamine-B and methylene blue solution passing through the AC pump side channel (Fig. 5b). At the initial state of room temperature, the two dye solutions separated into two opposite directions at the crossroads; the pink solution moved to the LIM, and the blue solution went to the AC pump (Fig. 5a). As the two dye solutions were drawn toward each side of the outlet, *f*_*b*_ is 1 and *f*_*a*_ is zero (Fig. 5c). After heating the pumps to a temperature higher than LCST, as the water extruded from the shrunken SIM decreased the pumping rate of the LIM, some of pink solution was drawn toward the AC pump whose pumping performance is stronger (Fig. 5b). Thus, *f*_*a*_ increased with the increase of *w*_*a*_ (Fig. 5c). After 120 s of heating, *f*_*a*_ and *f*_*b*_ became to have almost the same value of 0.5 ± 0.04. This implied that both solutions were almost equally drawn toward the AC pump. With cooling down to room temperature, the *f*_*a*_ decreased to zero which indicated that LIM starts to pump the solution again. These results show the strong potential of the LIM in various liquid-pumping devices with a special thermo-controlled function (Figures S5 and 6).

### Insulin pumping performance

**Figure 6:**
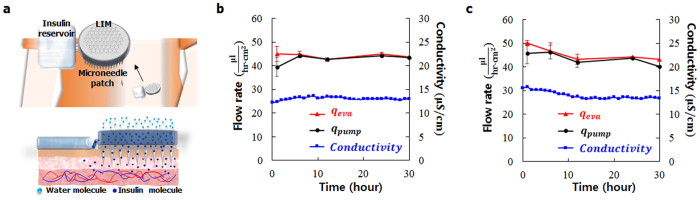
Pumping performance of the LIM for insulin solution. (**a**) A schematic illustration of implantable insulin pump as a potential application of the LIM. (**b,c**) The evaporation rate of water (*q*_*eva*_), pumping rate of IS solution (*q*_*pump*_), and the electrical conductivity inside the IS solution reservoir were measured for IS concentration of (**b**) 30 μM and (**c**) 90μM.

Based on the advantages of our LIM, we suggest possible application of the LIM for long term delivery of therapeutic agent (Fig. 6a). To examine the pumping performance for a therapeutic agent, the LIM was installed at the reservoir of insulin (IS) solution. The evaporation rate of water and pumping rate of IS solution by the LIM were measured continuously for 30 h at temperature of 24.89 ± 0.09 °C and relative humidity of 40.49 ± 1.16%. To analyze the effect of IS concentration on the pumping performance, two different IS concentrations, 30μM and 90μM, were tested. The concentration change in the IS solution reservoir was estimated by monitoring variation of its electrical conductivity.

In the 30 h continuous pumping of 30μM of IS solution by the LIM, the evaporation rate of water was 44.23 ± 0.85μl/h·cm^2^ and the pumping rate of IS solution was 42.86 ± 1.84μl/h·cm^2^ (Fig. 6b). During the experiment, the electrical conductivity inside the IS solution reservoir was consistently maintained at 13.05 ± 0.3μS/cm. This indicated that the IS concentration inside the reservoir did not change noticeably. When the IS concentration increased to 90μM, the evaporation rate of water was 45.47 ± 2.63μl/h·cm^2^ in average (Fig. 6c), and the IS solution pumping rate was 43.54 ± 2.31μl/h·cm^2^. For the higher IS concentration of 90μM, the electrical conductivity was higher than that of the lower IS concentration of 30μM (Figure S7). During the pumping performance test, the electrical conductivity inside the IS solution reservoir moderately maintained at 13.95 ± 0.77μS/cm in average. In addition, any crystallization of the IS solution was not observed during the experiment.

## Discussion

A novel non-mechanical micropump driven by evaporation, which is compact and portable, is developed in this study. On the basis of plant-leaf transpiration, we demonstrate that the proposed pump transports water upward against gravity by means of water potential difference. The highly water-permeable MAC easily delivers water from the water reservoir to the SIM in which negative water potential is generated by water evaporation. The pump can supply a liquid including therapeutic agent solution at a continuous pumping rate of around 2μl/min for over 30 h at room temperature.

The LIM has unique advantages of compact, portable and free from external mechanical or electrical power sources for operating the pump (Table 1). In addition, the pumping timing can be smartly manipulated. Based on these advantages of the LIM, we suggest a novel design of insulin pump integrated with a microneedle patch (Fig. 6a). As water evaporates from the LIM surface, insulin solution is pumped from the reservoir. Then the insulin molecules pumped from the reservoir of therapeutic drug are diffused into the skin through the patch of microneedles^30^. If the suggested pump is attached on a human body as schematically illustrated, it can guarantee a continuous and durable pumping rate, because the temperature of body surface is almost constant around 30 °C. If all the solute in the pumped solution is assumed to be injected to a patient, the LIM pump guarantees a stably dose of 1IU/h and 3IU/h, when IS solutions of 30μM and 90μM concentration are used, respectively.

The infusion rate can be increased by enlarging the size of the pump. If it is realized, the LIM can overcome the drawbacks of conventional drug-delivery pumps which are expensive and bothersome to handle due to their bulky size and the needs of external electical power supply. Without any electrical power source, the LIM offers epoch-making advantage in terms of power consumption in long-term usage. Moreover, the continuous supply of liquid at a ultra-slow flow rate for a very long period of time meets the requirements of continuous dosage of therapeutic drugs and long term on-demand release. However, we need to optimize the design of the pump for a specific application, so that the accumulated therapeutic drugs due to long-term use do not noticeably affect the pumping performance.

As the evaporation rate largely depends on temperature variation, a sudden change in temperature may induce over/under dose. Thus the LIM has to be operated in a temperature responsive manner, just in case. The pumping rate of water is thermally controlled by adapting a thermo-sensitive polymer membrane in a manner similar to the gating function of stomata in plant leaves. As the SIM shrinks and swells in response to temperature changes, the timing of water pumping is easily and smartly controlled. Therefore, the LIM system can resolve the technical limitations encountered in previous evaporative micropumps whose pumping rate is only passively changed as temperature varies (Table 1). On the other hand, the use of thermo-responsive hydrogel having relatively slow response at the specific LCST may have technological limitations in some applications where fast response of the pump is needed. The response time can be improved by adapting a very thin membrane with porous networks for increasing diffusion coefficient^31^. In addition, by shifting the LCST of the SIM or by utilizing other stimuli-responsive hydrogels which have faster responses to chemical, pH, light, or humidity, the pump can be further improved with more flexibility^32^,^33^.

In practical applications, the LIM can be utilized in a vast variety of fluid-based devices, such as POC systems and organ-on-a-chips to supply bio-samples. In scientific research, valuable or electrically sensitive specimens, including DNA molecules, cells with fragile membranes, and pharmaceutical drugs, would be delivered in a smart and precise manner without damaging by electrical or mechanical treatments.

## Methods

### Fabrication of LIM

A mold was fabricated with polylactic acid (PLA) using a 3D printer (MakerBot replicator 5th generation) to prepare the body of the micropump (Figure S1a). PDMS prepolymer was prepared by mixing a PDMS base and a curing agent at a weight ratio of 10:1. The PDMS prepolymer was poured onto the mold and then cured at 60 °C for 2 h (Figure S1b). The middle of the PDMS body was punctured, and a 15G needle was attached. The PDMS body and the needle were completely filled with 2% (w/v) agarose solution, frozen at −20 °C for 12 h, and melted at room temperature to prepare an agarose cryogel (Figure S1c). The agarose solution was also gelled at room temperature to prepare a normal agarose gel. The 2% concentration of agarose gel is appeared to guarantee high water permeability through the gel and to maintain the mechanical structure not to be too brittle.

Photo-crosslinkable PNIPAAm was synthesized through free radical polymerization to fabricate the SIM (Figure S1d). A pre-gel solution was prepared by dissolving 100 mg of *N*-isopropylacrylamide monomer (NIPAAm, Sigma Aldrich) in 0.7 ml of deionized water. Subsequently, *N*,*N*′-methylenebisacrylamide (MBAm; Sigma Aldrich; 1 mg for the LC SIM, 3 mg for the HC SIM) and 1 mg of 2-hydroxy-1-[4-(hydroxyethoxy)phenyl]-2-methyl-1-propanone (Irgacure 2959; Sigma Aldrich) were added to the monomer solution as a crosslinker and a photoinitiator, respectively. The pre-gel solution was covered with a transparent photomask on which the slit patterns were printed, and then irradiated with UV-light (VIRVER Lourmat-4.L, France; *λ* = 365 nm, 4 W) for 4 min. These processes were sequentially conducted, as in our previous study^27^. The fabricated SIM was carefully peeled off from the photomask and applied to the agarose cryogel of the pump. The edge of the SIM was anchored to the pump with a glue (Figure S1e).

### Characteristics of SIM

The surface morphologies of the fabricated SIMs were observed under a stereoscope (Stemi 2000c; Zeiss, Germany) attached to a digital camera (NIKON D700, Tokyo, Japan). The morphological structures of mesophyll cells and the surface characteristics of the SIMs were examined through field-emission scanning electron microscopy (XL30S FEG FE-SEM, Phillips). The swelling ratio based on the weight of the SIM was defined as follows: (*m*_*f*_ − *m*_*i*_)/*m*_*i*_, where *m*_*f*_ and *m*_*i*_ are the final and initial masses of the membrane, respectively.

### Measurement of flow rate

The flow rate was measured by using the following equation: (*M*_*f*_ − *M*_*i*_)/*t*, where *M*_*f*_ and *M*_*i*_ denote the final and initial masses, respectively, and *t* is the time interval of mass measurements. The evaporation rate was estimated by measuring temporal mass variations of the pump system and the water reservoir with a microbalance (CAS, CAUW 220D). Except the evaporative surface of the pump, all other parts were sealed to prevent unexpected water loss, so that the water loss in the reservoir can directly represent the amount of water evaporation. For measuring the pumping rate, the pump was carefully unloaded from the water reservoir, and the mass variations in individual reservoirs were measured.

In general, evaporation rate is largely dependent on environmental conditions surrounding the evaporative surface, including temperature, relative humidity, pressures, and flow rate of air. As only the temperature effect on the pumping performance was investigated in this study, all other factors except temperature were used as control variables during the experiment. Thus, the experiment was conducted inside an air conditioned room where temperature and humidity were maintained at 25 °C and 40%, respectively. For conducting the heating experiment of the pump, only the pump was locally heated using an electric heater.

To analyze the pumping performance of therapeutic agents, an insulin recombinant human (Sigma-Aldrich) dissolved in DI water was used. The electrical conductivity of the insulin solution was measured using a conductivity meter (Vernier Lab Quest with electro-probe).

### X-ray micro-imaging

An X-ray imaging experiment was conducted at the 6C (Bio-medical imaging) beamline of Pohang Light Source (PLS-II). Real-time phase-contrast X-ray images were consecutively recorded with intervals of 1 s by using a CCD camera (PCO 4000) equipped with a 5× objective lens with a field of view (FOV) of approximately 7,200 × 4,800 μm^2^ with 1.8 μm of pixel size. The corresponding spatial resolution was approximately 5 μm/pixel based on the pixel size of the camera. The distance from the test sample to the camera was 20 cm. A 1 mm thick silicon wafer was positioned in the pathway of the X-ray beam to minimize the photo-thermal damage to the test samples. The consecutively recorded X-ray images revealed the temporal evolution of water transport phenomena in the test sample.

The 3D morphological structures of agarose gel and agarose cryogel were observed through X-ray micro-computed tomography. A 4× objective lens was attached to the front of a sCMOS camera (Andor Zyla, Belfast, UK) with a 2560 × 2160 pixel resolution. The spatial resolution was approximately 1.6 μm/pixel for the FOV of 4.2 mm × 3 mm. An experimental model was attached to the sample holder, which was then rotated from 0° to 180° at an interval of 1°. The 3D morphological structures were numerically reconstructed using Octopus (inCT) and rendered using Amira® (Visualization Sciences Group).

### Fabrication of microchannels

An acrylic mold was carved into an H-shape with a depth of 150 μm by using a milling machine. Agarose gel (3%) was poured over the mold and cured. The cured agarose gel was then used as the mold for the PDMS prepolymer. The degassed PDMS prepolymer was poured on the agarose gel mold and cured at 60 °C for 90 min. Afterward, the PDMS channel was peeled off and attached to another PDMS membrane with a thickness of 1 mm.

## Additional Information

**Publisher's note**: Springer Nature remains neutral with regard to jurisdictional claims in published maps and institutional affiliations.

**How to cite this article**: Kim, H. *et al.* Compact and Thermosensitive Nature-inspired Micropump. *Sci. Rep.* **6**, 36085; doi: 10.1038/srep36085 (2016).

## Supplementary Material

Supplementary Movie S1

Supplementary Movie S2

Supplementary Movie S3

Supplementary Movie S4

Supplementary Movie S5

Supplementary Movie S6

Supplementary Information

## Figures and Tables

**Figure 1:**
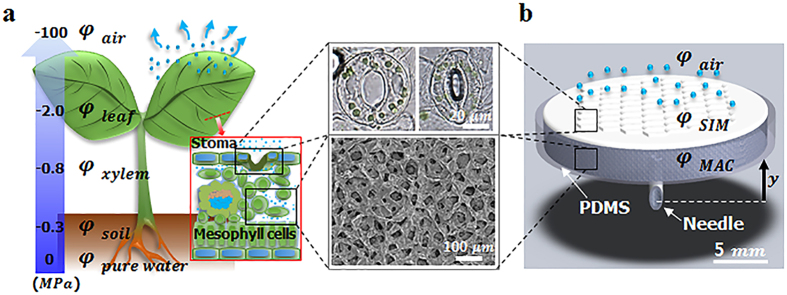
Schematic of a plant leaf and a leaf-inspired micropump (LIM). (**a**) In plants, water is transported against gravity from roots to leaves via a water potential gradient. Mesophyll cells inside leaves exhibit high water permeability for easy water transport. Stomata function as a hydraulic valve to control gas exchange. (**b**) The morphological structure of LIM is analogous to that of a plant leaf. The pump is composed of a SIM and a MAC. Water is delivered from the needle to the SIM via the MAC against gravity with the aid of a water potential gradient.

**Table 1:** Specifications of water evaporation driven micropumps.

Reference number (year)	Power-less	Flow rate (µl/hr)	Thermo-reactivity	Active flow control (method)	Operating time	Portable	Applicable
Kim *et al.* (Our device)	o	30	Active	o (Heating)	Days	o	o
14 (2002)	o	14.4~25	Passive	o (Airflow)	—	x	x
15 (2002)	o	0.6–60	Passive	x (Sorption agent)	Days	o	o
16 (2002)	o	2.9 (P), 72 (A)	Passive	x	Single use	o	x
17 (2003)	o	0.3	Passive	o (Heating)	∼2 hrs	x	x
18 (2005)	x	0.1–4.3	Passive	o (Heating, peltier electrode)	—	x	x
19 (2006)	o	180	Passive	x	Few hrs	o	o
20 (2008)	o	32.4–140	Passive	x	~400 hrs	x	o
21 (2009)	o	0.03	Passive	x	~6 hrs	o	o
22 (2011)	o	0.24~0.48 (P), 6.8~7.6 (A)	Passive	o (Heating)	—	o	X
23 (2014)	x	84.6	Passive	o (Piezoelectric)	—	x	x
24 (2015)	o	0.4~7.2	Passive	x	Single use	o	x

P, passive control; A, active control.
