# Supplementary material for: Compact and Thermosensitive Nature-inspired Micropump
Source: Sci Rep. 2016 Oct 31;6:36085. doi: 10.1038/srep36085 (PMC5086846; doi:10.1038/srep36085)
Supplement: Supplementary Information [file srep36085-s7.pdf]

# Supporting Information

## Compact and Thermosensitive Nature-inspired Micropump

Hyejeong Kim[1] , Kiwoong Kim[1] and Sang Joon Lee[1,*]

**CONTENTS**

**Supplementary Fig. 1** Fabrication procedure of the LIM.

**Supplementary Fig. 2** Liquid permeability difference of agarose gel and agarose cryogel.

**Supplementary Fig. 3** Water content inside the SIMs.

**Supplementary Fig. 4** Comparison of the thermo-responsive performance.

**Supplementary Fig. 5** LIM and AC pump are integrated into an H-shaped channel.

**Supplementary Fig. 6** Integration of three pumps into the microchannel.

**Supplementary Fig. 7** Electrical conductivity of insulin solution.

**1. Theoretical estimation of evaporation rate of the SIM**

**1.1. Effect of temperature on evaporation rate**

**1.2. Effect of relative humidity on evaporation rate**

**1.3. Thermo-controlled pumping procedure of the micropump**

**2. References**

**Supplementary Movie 1.** Water absorption by agarose cryogel.

**Supplementary Movie 2.** Opening slits.

**Supplementary Movie 3.** Water extrusion from flat PNIPAAm membrane.

**Supplementary Movie 4.** Water extrusion from the SIM.

**Supplementary Movie 5.** Water extrusion from the SIM, absorption by agarose cryogel.

**Supplementary Movie 6.** Flow control experiment with AC pump and LIM pump.

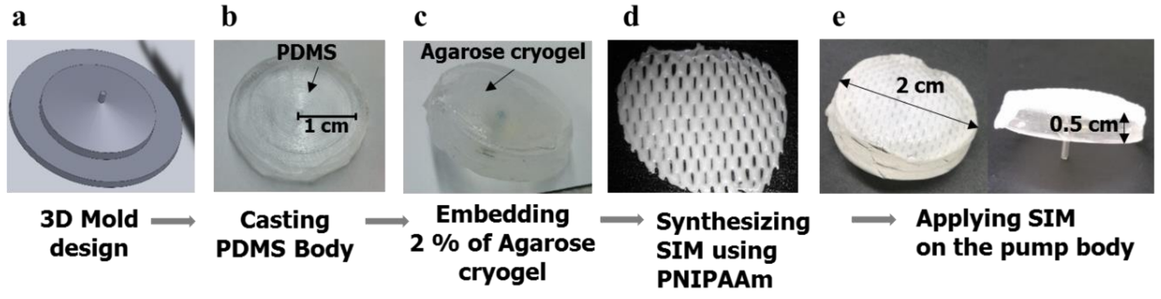


**Supplementary Fig. 1** Fabrication of the LIM. (a) A mold was fabricated by using a 3D printer to prepare the micropump body. (b) A PDMS prepolymer was casted onto the mold and cured at 60 °C for 2 h. (c) The middle part of the PDMS body was punctured, and a needle was attached. The pump filled with 2% agarose gel was frozen at −20 °C for 12 h and melted at room temperature to prepare an agarose cryogel. (d) Photo-crosslinkable poly(*N*-isopropylacrylamide) was synthesized through free-radical polymerization to fabricate the SIM. (e) The SIM was applied to the agarose cryogel and the edge of the SIM was anchored to the pump by a glue.

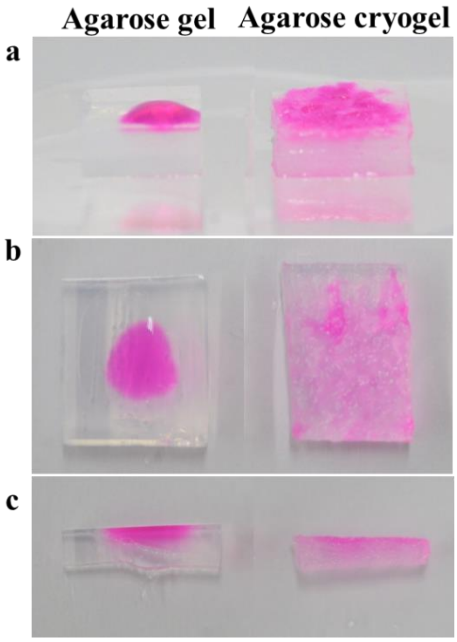


**Supplementary Fig. 2** Comparison of the liquid permeability difference between nanoporous agarose gel and macroporous agarose cryogel by dropping Rhodamine-B solution on the surface of each gel. (a) Side view of the initial state. Rhodamine-B solution spreads on the hydrophilic surface of the agarose gel but does not permeate vertically. The dye solution percolates into the agarose cryogel. (b) Top view of the initial state. The solution does not spread radially on the agarose gel, but the solution radially spreads further on the agarose cryogel. (c) After 10 min, Rhodamine-B solution vertically penetrates the gel.

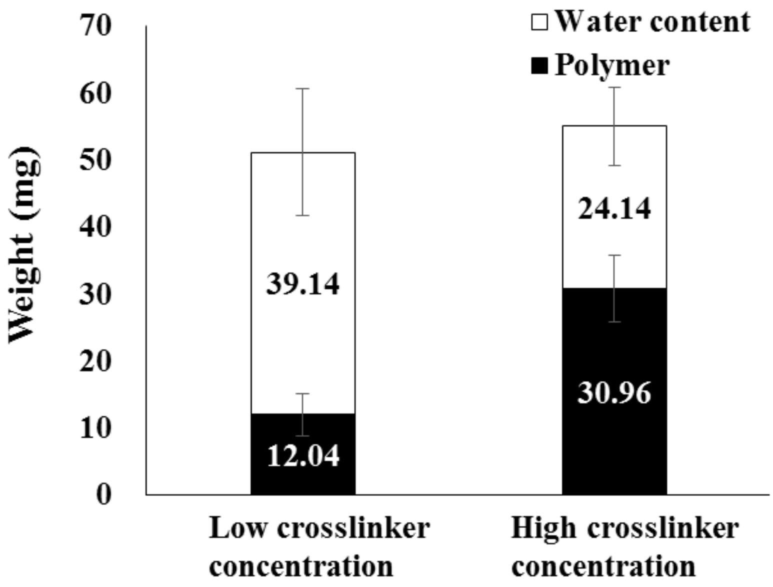


**Supplementary Fig. 3** Water contents inside the SIMs when they fully swell. The SIM containing a low of crosslinker concentration is composed of high amounts of water because the high concentration of the crosslinker in the pregel solution induces high polymerization degree and low water swelling. In particular, 76.5% and 43.8% of the LC SIM and the HC SIM consist of water.

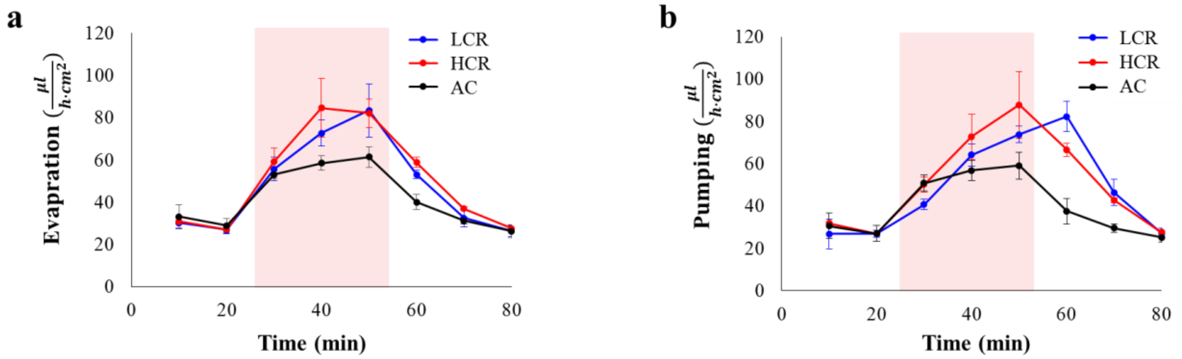


**Supplementary Fig. 4** Comparison of the thermo-responsive performances of AC, LC LIM, and HC LIM. (a) Variations of the average evaporation rates of the pumps. At room temperature, the three pumps exhibit similar evaporation rates. LIMs evaporate a higher amount of water than the AC pump does when the LIMs are heated to temperatures higher than LCST. This phenomenon is attributed to the bumpy surface of the SIMs and thus increases the evaporative surface area. (b) Variations of the average pumping rate of the pumps. The pumping performance of the LC LIM is delayed to a greater extent than that of the HC LIM.

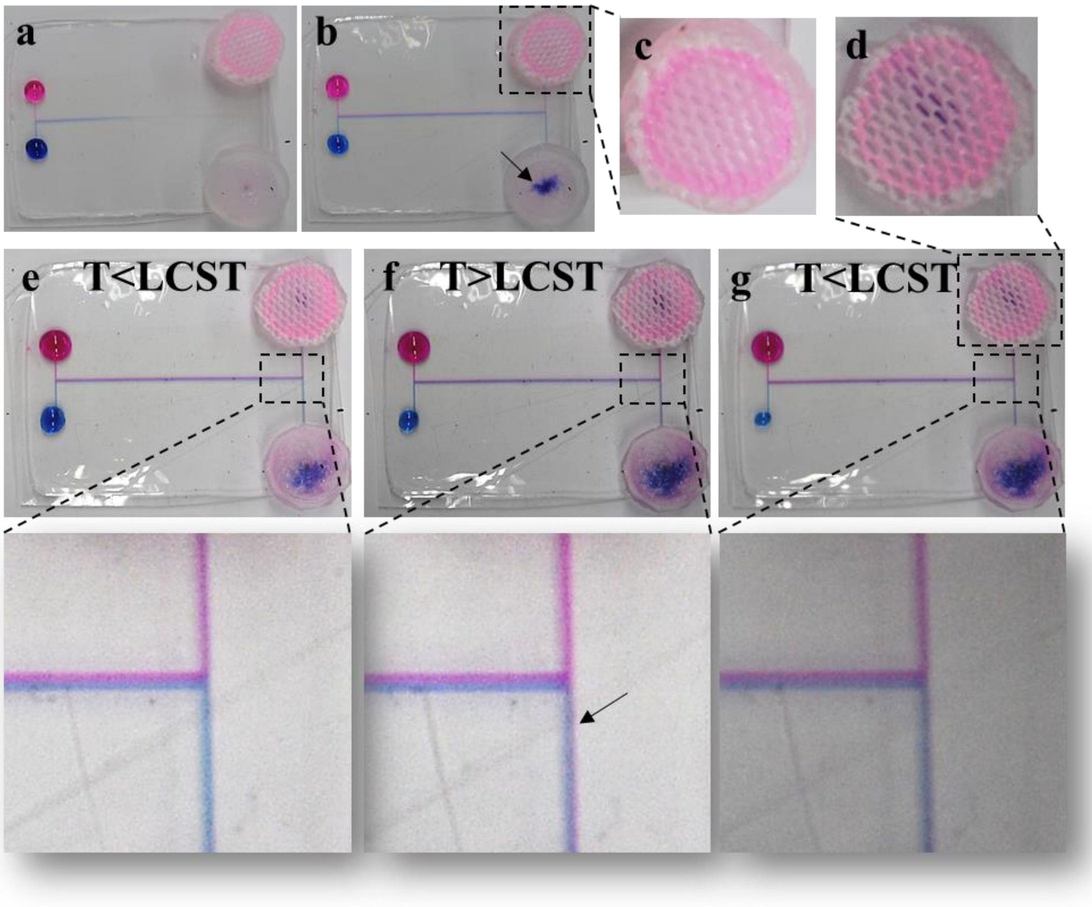


**Supplementary Fig. 5** LIM and AC pump are integrated into an H-shaped channel. (a) Dye solutions of Rhodamine-B and methylene blue are dropped at the inlet sides of the LIM and the AC pump, respectively. (b) As water evaporates, the two dye solutions are drawn toward the outlet sides of the pumps. (c) Initial state of the SIM surface. (d) Final state of the SIM surface after it was heated at a temperature higher than LCST. (e) At room temperature, the two dye solutions are separated toward opposite directions at the crossroads; the pink solution moves to the SIM and blue solution goes to the AC pump. The dotted square region of the image is enlarged for clarity. (f) After the pumps are heated to a temperature higher than LCST, the pink and blue solutions are drawn toward the AC pump. (g) After the pumps are cooled to a temperature below LCST, the two dye solutions are separated again into opposite directions.

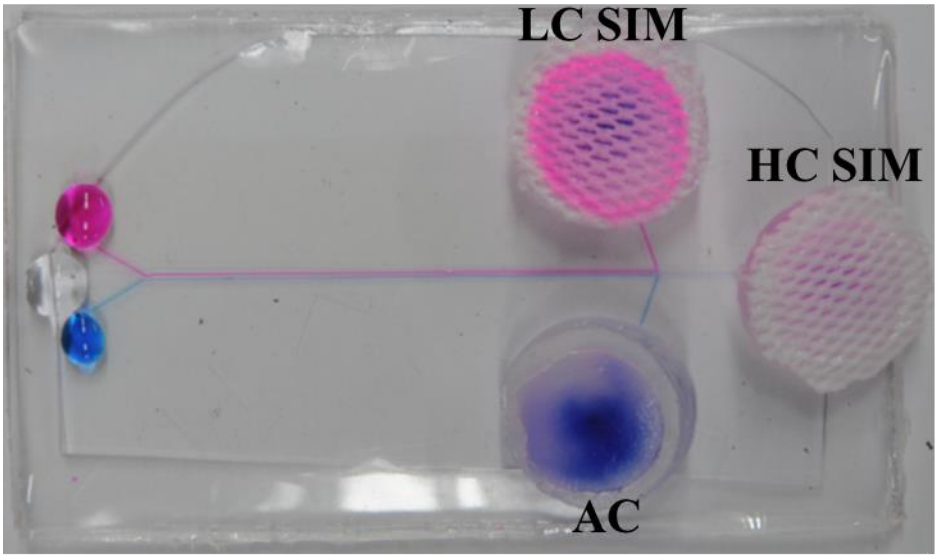


**Supplementary Fig. 6** Integration of AC pump (blue), LIM with LC SIM (pink), and LIM with HC SIM (white) into the microchannel. The compact and portable LIM can be easily applied in various liquid pumping systems with thermo-controlled function.

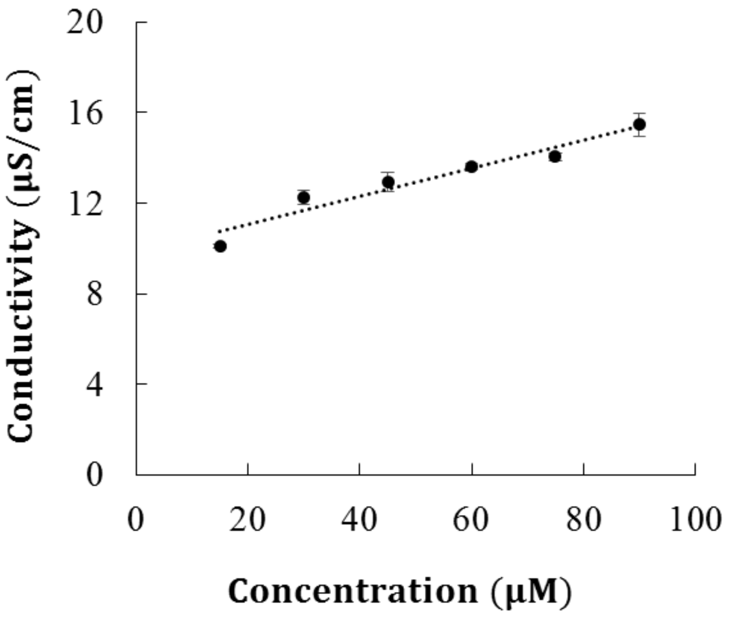


**Supplementary Fig. 7** Electrical conductivity of insulin solution according to the concentration variation.

## 1. Theoretical estimation of the evaporation rate of the SIM

Evaporation occurs on the surface of the hydrophilic SIM with a contact angle of 71.1° $\pm$ 6.3° and on the exposed area of the MAC through the slit pores of the SIM when the LIM is exposed to a temperature lower than LCST. The pores of the SIM open as the material shrinks and the surface becomes hydrophobic with a contact angle of 90.1° $\pm$ 1.1° when the LIM is heated at a temperature higher than LCST. Evaporation also occurs at the hydrophobic SIM and the exposed area of the hydrophilic MAC through the open pores of the SIM.

The evaporation rate can be expressed as follows: $q_{eva} \sim \exp(0.0566 \cdot T) \cdot f(\theta) \cdot S$ (**eq 1**), where $T$ is the absolute temperature of vapor, $f(\theta)$ is the function of the contact angle $\theta$ of water vapor,[1, 2] and $S$ is the area of the evaporative surface. The wettability of the evaporative surface, which is changed by temperature variation, determines the value of $f(\theta)$. The deformability of the SIM determines the area of the evaporative surface. Therefore, the total evaporation rate on the SIM surface exhibits combined changes with respect to $T$, $f(\theta)$, and $S$ as temperature changes. For instance, the evaporation rate can be estimated as $q_{eva} \sim 0.69 \cdot S_t \cdot \exp(0.0557 \cdot T)$ at temperatures lower than LCST when the LC SIM is applied to the pump. At temperatures higher than LCST, the evaporation rate is $q_{eva} \sim 0.72 \cdot S_t \cdot \exp(0.0557 \cdot T)$, where $S_t$ denotes the total surface area of the SIM.

The evaporation rate on the surface of the SIM can be expressed as follows:

$q_{eva} = -2\frac{\pi D M a}{\rho}[c(T_w) - Hc(T_a)]f(\theta)S.$ (**eq 2**), [3]

where $D$ is the diffusivity of vapor at ambient temperature, $a$ is the radius of the bounding circle, $M$ is the molecular mass of vapor molecules, $H$ is the relative humidity, and $c(T_w)$ and $c(T_a)$ are the saturated water vapor molar concentration in air at the liquid–gas interface and ambient, respectively.

According to ideal gas law, the saturated vapor concentration follows the relationship of $c(\mathrm{T}) = \frac{1}{RT}\exp\left(20.386 - \frac{5132}{T}\right) \approx \frac{6\cdot10^{-9}}{R}\exp(0.0526\cdot T)\, mmHg$ .[2] Thus, **Eq. (2)** can be estimated as follows:

$$q_{eva} \approx -1.2\times10^{-8}\cdot\frac{\pi DMa}{\rho R}\{\exp(0.0526\cdot T_w) - H\cdot\exp(0.0526\cdot T_a)\}\cdot f(\theta)\cdot S.$$ (**eq.(3)**)

Through experimental estimation, $H$ is expressed as $H \sim -1.48T + 84.5$ at 293 K < $T$ < 318 K. Assuming that the temperature at the liquid-gas interface and the ambient are the same ($T_w \approx T_a = T$), we can estimate **eq. (3)** as follows:

$q_{eva} \approx k\cdot\exp(0.0566\cdot T)\cdot f(\theta)\cdot S$ (**eq.1**), where $k = -1.3\cdot10^{-6}\cdot\frac{\pi DMa}{\rho R}$.

The contact angle of the PNIPAAm membrane can be estimated by using the experimental value as a function of temperature. Then, $\mathrm{f}(\theta)$ can also be estimated as a function of temperature: $\mathrm{f}(T) \sim 0.0033(T+188)$. For the hydrophilic agarose cryogel, $\mathrm{f}(\theta)$ is almost constant: $\frac{2}{\pi}\sim 0.63$. $f(\theta)\cdot S$ is estimated using the following equation: $(f(\theta)\cdot S)_{tot} = (f(\theta)\cdot S)_{SIM} + (f(\theta)\cdot S)_{Pore}$.

| **Pump wo/ SIM** | $\mathbf{f(\theta)}$ | S | $\boldsymbol{f(\theta)\cdot S}$ | $\boldsymbol{(f(\theta)\cdot S)_{tot}}$ |
|---|---|---|---|---|
| T | 0.63 | $S_t$ | $0.63\cdot S_t$ | $0.63\cdot S_t$ |

| **Pump with LCR SIM** | | $\mathbf{f(\theta)}$ | S | $\boldsymbol{f(\theta)\cdot S}$ | $\boldsymbol{(f(\theta)\cdot S)_{tot}}$ |
|---|---|---|---|---|---|
| $T$ < LCST | SIM | 0.7 | $0.92\cdot S_t$ | $0.64\cdot S_t$ | $0.69\cdot S_t$ |
| | Pore | 0.63 | $0.08\cdot S_t$ | $0.05\cdot S_t$ | |
| $T$ > LCST | SIM | 0.74 | $0.81\cdot S_t$ | $0.60\cdot S_t$ | $0.72\cdot S_t$ |
| | Pore | 0.63 | $0.19\cdot S_t$ | $0.12\cdot S_t$ | |

| **Pump with HCR SIM** | | $\mathbf{f(\theta)}$ | S | $\boldsymbol{f(\theta)\cdot S}$ | $\boldsymbol{(f(\theta)\cdot S)_{tot}}$ |
|---|---|---|---|---|---|
| $T$ < LCST | SIM | 0.7 | $0.96\cdot S_t$ | $0.67\cdot S_t$ | $0.70\cdot S_t$ |
| | Pore | 0.63 | $0.04\cdot S_t$ | $0.03\cdot S_t$ | |
| $T$ > LCST | SIM | 0.74 | $0.85\cdot S_t$ | $0.63\cdot S_t$ | $0.72\cdot S_t$ |
| | Pore | 0.63 | $0.15\cdot S_t$ | $0.09\cdot S_t$ | |

$S$ was evaluated on the basis of the measured pore surface area in Figure 3b.

### 1.1. Effect of temperature on evaporation rate

From (eq.2), by assuming that all other factors are constant, the evaporation rate is exponentially increased as temperature increases.

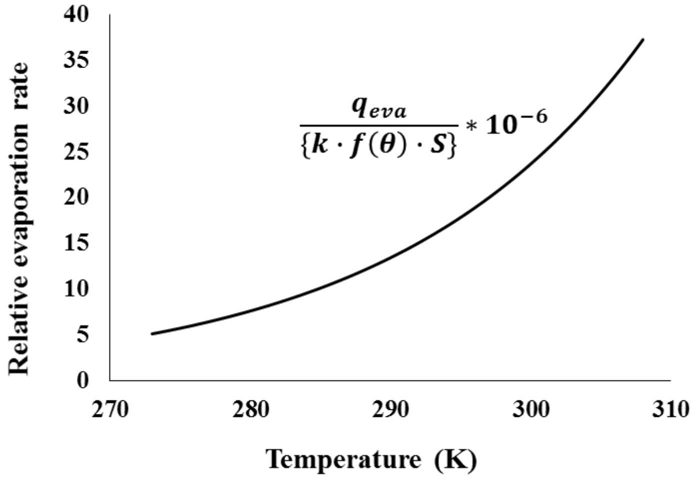


### 1.2. Effect of relative humidity on evaporation rate

From (eq.2), by assuming that all other factors are constant, the evaporation rate is linearly decreased as relative humidity is decreased, where $m = -2 * \frac{\pi DMa}{\rho}$.

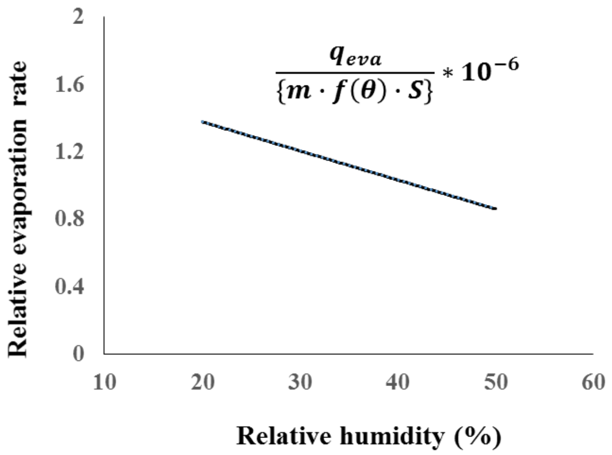

### 1.3. Thermo-controlled pumping procedure of the micropump

The pumping rate of the pump is affected by the combined effect of water evaporation and swelling/deswelling features of the material composing the SIM. Therefore, we divided the evaporation of water in the SIM and the pumping of water from reservoir into four stages based on the variation and duration of temperature.

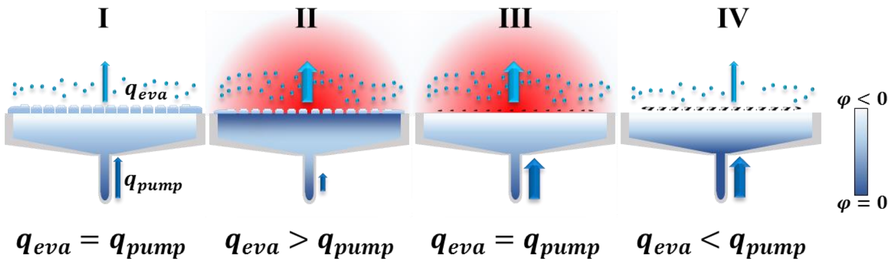


**Figure R4.** Thermo-controlled pumping procedure of the micropump.

i. T < 33℃ (1st stage)

The evaporation flow rate and the pumping flow rate are almost the same. The evaporation-induced water loss in the SIM is compensated by the water pumped from the reservoir

$$q_{pump} = q_{eva} \approx k \cdot \exp(0.0566 \cdot T) \cdot f(\theta) \cdot S.$$ (eq.1 in supporting information 1)

ii. T > 33℃ (2nd stage)

When the temperature is higher than LCST, the pumping rate has a relationship with evaporation rate and water extrusion from the SIM as below.

$$\int_0^t q_{pump} = \int_0^t \int_{33°C}^T q_{eva}(T)\, dT \cdot dt - \int_0^t \int_{33°C}^T q_{extrusion\ from\ SIM}(T)\, dT \cdot dt.$$

where t is the duration time during which the temperature is higher than 33℃, and $q_{extrusion\ from\ SIM}$ is the water extrusion rate from the SIM. Following the relations explained in supporting information 1.1, evaporation rate, $q_{eva}$, is the function of temperature T. As the water extruded from the SIM is absorbed at the MAC, $\varphi_{MAC}$ increases with reducing $\Delta\varphi$ between the MAC and the water reservoir. When the total evaporation from the pump exceeds the water extrusion from the SIM, the flow rate of the pump is smaller than the evaporation rate.

iii. T > 33℃, (3rd stage)

When the temperature is maintained higher than 33℃, all of the absorbed water from THE SIM evaporates. Thus, the pumping rate and the evaporation rate become almost the same once again as

$$q_{pump} = q_{eva} \approx k \cdot \exp(0.0566 \cdot T) \cdot f(\theta) \cdot S.$$

iv. T < 33℃ (4th stage)

When the temperature is decreased lower than 33℃, the pumping rate has a following relationship for the evaporation rate and water absorption by the SIM.

$$\int_0^t q_{pump} = \int_0^t \int_{33°\mathrm{C}}^T q_{eva}(T)\, dT \cdot dt + \int_0^t \int_{33°\mathrm{C}}^T q_{absorption\ of\ SIM}(T)\, dT \cdot dt.$$

where t is the duration time during which the temperature is lower than 33℃, and $q_{absorption\ by\ SIM}$ is the water absorption rate of the SIM. As temperature decreases lower than LCST, the SIM becomes to have hydrophilic feature and absorbs water. Therefore, the pumping flow rate of water is the sum of the evaporation flow rate and the water absorption rate of the SIM.

## 3. References

[1] F. Schönfeld, K.H. Graf, S. Hardt, H.J. Butt, *Int. J. Heat Mass Transfer* 2008, 51, 3696.

[2] J. D. Current, *Physics Related to Anesthesia*, PediaPress, 2011.

[3] C. Nie, A. J. Frijns, R. Mandamparambil, J. M. den Toonder, *Biomed. Microdevice*. 2015, 17, 1.
